# Laser-based three-dimensional manufacturing technologies for rechargeable batteries

**DOI:** 10.1186/s40580-021-00271-w

**Published:** 2021-08-09

**Authors:** Dan Moldovan, Jaeyoo Choi, Youngwoo Choo, Won-Sik Kim, Yoon Hwa

**Affiliations:** 1 The School of Electrical, Computer and Energy Engineering, Arizona State University, Tempe, AZ 85281 USA; 2 The Molecular Foundry, Lawrence Berkeley National Laboratory, Berkeley, CA 94720 USA; 3 Materials Sciences Division, Lawrence Berkeley National Laboratory, Berkeley, CA 94720 USA; 4 Department of Materials Science and Engineering and Research Institute of Advanced Materials, Seoul National University, Seoul, 151-744 Republic of Korea

**Keywords:** Rechargeable batteries, Three-dimensional printing, Laser manufacturing, Additive manufacturing, Subtractive manufacturing

## Abstract

Laser three-dimensional (3D) manufacturing technologies have gained substantial attention to fabricate 3D structured electrochemical rechargeable batteries. Laser 3D manufacturing techniques offer excellent 3D microstructure controllability, good design flexibility, process simplicity, and high energy and cost efficiencies, which are beneficial for rechargeable battery cell manufacturing. In this review, notable progress in development of the rechargeable battery cells via laser 3D manufacturing techniques is introduced and discussed. The basic concepts and remarkable achievements of four representative laser 3D manufacturing techniques such as selective laser sintering (or melting) techniques, direct laser writing for graphene-based electrodes, laser-induced forward transfer technique and laser ablation subtractive manufacturing are highlighted. Finally, major challenges and prospects of the laser 3D manufacturing technologies for battery cell manufacturing will be provided.

## Introduction

Electricity has been a bloodline of modern society and one cannot think of our life without it. Electricity is produced at centralized power plants with various energy sources, such as fossil fuels and renewable energy sources, and then delivered to consumers. This network is often referred to as the electric power grid, and the use of electronics for our daily lives used to strongly rely on the electric power grid until rechargeable batteries, particularly lithium (Li) ion batteries, were commercialized by the Sony and Asahi Kasei teams led by Nishi in 1991 [1]. The Li-ion battery is a contemporary example of a rechargeable battery that revolutionized our lives by enabling the use of portable electronics such as cell phones, laptops, and power tools without a power cable. More recently, the market of rechargeable batteries has expanded from small mobile electronics into larger applications such as electric transportation and energy storage systems for power grids, and rechargeable batteries have been regarded as a major contributor to a fossil fuel-free future. However, the emerging applications require significantly improved energy and power density, fast charging capability, and excellent safety with affordable cost, which is unlikely fulfilled by the conventional rechargeable batteries such as Li-ion and nickel metal hydride cells. For this reason, enormous research effort has still been dedicated to developing advanced rechargeable batteries.

Rechargeable batteries are generally composed of one or more electrochemical cells which store electrical energy in the form of chemical energy (charging the battery) and convert the chemical energy back to electricity when the battery is being used. In the electrochemical cells, two electrodes with different chemical potentials are electronically insulated but ionically connected through an ion reservoir called electrolyte, which may be a liquid, gel, or solid. The electrode with lower potential is often called the anode and the electrode with higher potential is referred to as the cathode in the battery industry. Although the definitions of the anode and the cathode are slightly different from those in traditional electrochemistry, they are generally accepted by the users of rechargeable batteries, so they will be used in this review.

The theoretical maximum energy storage capability of the rechargeable cells (Wh/kg or Wh/L) is determined by operating voltages (V) and capacities (Ah/kg or Ah/L) of the anode and the cathode. However, experimentally measured performance of the rechargeable cells is generally deviated from their theoretical maximum because physicochemical, electrical, and topological terms of the battery cell components can slow down electrochemical reaction kinetics, decreasing capacity or operating voltage of the rechargeable cells. To minimize the gap between theoretical maximum and practically obtainable performance of the battery cells, a remarkable advancement in the materials development has progressed by discovering new functional materials or modifying materials’ physicochemical and electronic properties. The advancement in material development will not be discussed in this review, but there are many review articles available [2–11]. While the development of functional materials is still necessary, significant effort into the structural design of the battery cell components needs to be dedicated in order for the rational design of electrode/electrolyte interface that is favorable for fast electrochemical process. For example, the conventional Li-ion cells employ porous and multi-component electrodes consisting of an active material and functional additives such as an electronic conductive carbon and a polymeric binder. When the Li-ion cell is assembled, liquid electrolyte is injected into the electrodes and fills the internal pores of the electrodes. The interconnection of the electrolyte-filled pores serves as an ion transport pathway while the interparticle connection serves as an electron transport pathway, implying that the microstructure of the electrode that determines electronic and ionic pathways should play a key role to improve electrochemical performance of the rechargeable cells [12].

Among electrode fabrication methods, the slurry-based wet processing has been widely used for the metal ion rechargeable cells such as Li-ion, lithium/sulfur (Li/S), sodium (Na) ion, and magnesium (Mg) ion cells that have actively been investigated for emerging applications [13]. Briefly describing the slurry-based wet fabrication process, solid electrode constituents are added to polymer binder solution and homogeneously mixed to form a slurry. The prepared slurry is cast onto a metallic current collector and dried, which results in a two-dimensional (2D) electrode film with a porous internal structure. The thickness of the electrode film can be controlled to some degree, but the internal pore structure relies completely on the physicochemical properties of the solid constituents and the evaporation of the solvent, so they cannot be precisely controlled. There have been various approaches to mitigate the drawbacks by optimizing physicochemical property of the slurry [14–17], slurry drying process parameters [18–21], and post-processing such as calendaring [22–24] and thermal treatment [25–27], but their effectiveness in electrode microstructure control is obviously limited.

In addition, one of the biggest challenges in the development of next-generation rechargeable cells is to achieve a much higher specific energy (Wh/kg) than that of the state-of-the-art of Li-ion cells, which requires thicker electrodes than that of conventional electrodes while still maintaining good electrochemical performance. However, as the electrode thickness increases, the distance of ion transport from bulk electrolyte to the active material near the bottom of the electrode inevitably increases and it is possible the ionic pathway becomes more complex, resulting in a slow electrochemical reaction rate. To improve the electrochemical performance of the thick electrodes, several approaches such as the sacrificial template method [28–37] and foam-like structured 3D current collectors [38–41] that modify the microstructure of the thick electrodes have been investigated, and notable improvements in cell performance were reported. However, those methods still lack an opportunity to precisely build the intended microstructure of the electrodes. The ice-templating methods such as freeze casting technique [29, 30, 35], and freeze tape casting technique [36, 37] have shown their excellent ability to build a 3D aligned microstructure of the electrodes or the solid electrolytes. However, some technical difficulties such as long processing time, the requirement of ultra-high vacuum for removal of the ice-template, and limited selection of processing solvent are the practical hurdles that prevent their widespread applications.

Recently, advanced laser manufacturing methods have attracted enormous attention because of their exceptional ability to fabricate 3D objects with complex net-shaped geometries. A 3D virtual model of a 3D object is designed by a computer-aided design (CAD) facility, and the designed 3D virtual model is then translated into motion control commands to execute a 3D printing process. High energy lasers are a popular energy source for 3D manufacturing because a focused laser beam can deliver a large amount of energy to the designated micro-scale focal region and induce a rapid photochemical reaction or photothermal phase transformation of printing materials. A photochemical reaction is often initiated by the dissociation of chemical bonds upon the irradiation of laser which induces subsequent chemical reaction driven by radicals. Hence, it is broadly employed in organic synthesis or crosslinking of the coated precursor materials in local area. The photothermal phase transformation utilizes rapid increase of temperature in focal area which induces oxidation of the precursor materials and thus, it is well adopted in sintering of inorganic materials or zone annealing. The laser manufacturing technologies enable high-precision 3D printing for a wide range of materials such as metals, ceramics, and organic compounds. The absorption characteristics of a material strongly depends upon the wavelength (near infrared, visible and ultraviolet radiation) and power level of the laser. Therefore, appropriate selection of the laser is necessary for the target material to ensure an efficient laser manufacturing process [42].

Various types of laser man ufacturing methods have recently been investigated for the manufacturing of rechargeable cells. Some of the methods used in the bottom-up approach are often referred to as laser additive manufacturing, while other methods used in the top-down approach are regarded as laser subtractive manufacturing. In the laser additive manufacturing (LAM), a focused laser beam intends to selectively sinter or melt material feedstock to build a 3D structure in a layer-by-layer method, whereas laser subtractive manufacturing (LSM) aims to selectively remove materials from a workpiece to create 3D feature without thermal damage to the surrounding. In this review, we introduce various laser manufacturing methods that are adopted in electrochemical batteries and discuss their notable achievements. Lastly, a brief summary and outlook of laser 3D manufacturing techniques for rechargeable cells will be provided.

## Selective laser sintering and selective laser melting techniques

Additive manufacturing (AM) is a computer-controlled process in which 3D CAD model of a printing object is sliced into many layers and built layer-by-layer using a proper printing tool. As its name implies, AM builds a 3D structure by adding material, opposite of traditional milling or machining methods, which remove materials to create a 3D object. Because a 3D object is printed in a layer-by-layer fashion, AM can construct complex and hollow internal structures without supporting materials, which is unlikely achieved by conventional manufacturing methods. In general, AM techniques used to be performed for rapid prototyping of structural parts, but it has recently been investigated for actual applications such as mass-customized parts, aerospace and biological devices, and automotive parts that require common characteristics with complex internal structures.

AM techniques have a significant potential to create a new paradigm in battery cell manufacturing because of their ability to construct complex “inner” porous microstructure of a 3D printing object. This is a great benefit of using AM in battery cell manufacturing because rational design of 3D porous microstructure of the battery cell components such as electrodes and solid electrolytes is a key strategy to improve performance and reliability of the battery cells. Among various AM techniques such as inkjet printing [43–45], stereolithography [46–48], material extrusion [49, 50], powder bed fusion (PBF) [43, 51, 52], directed energy deposition (DED) [53, 54], direct laser writing (DLW) for graphene patterns [55, 56], and laser-induced forward transfer (LIFT) [57, 58], we will focus on laser-based AM techniques that build 3D structures of printing objects using photothermal or photochemical effect of laser light. Research papers that adopt selective powder processing methods will mainly be reviewed in this section and some other LAM techniques will also be discussed in the following sections.

**Fig. 1:**
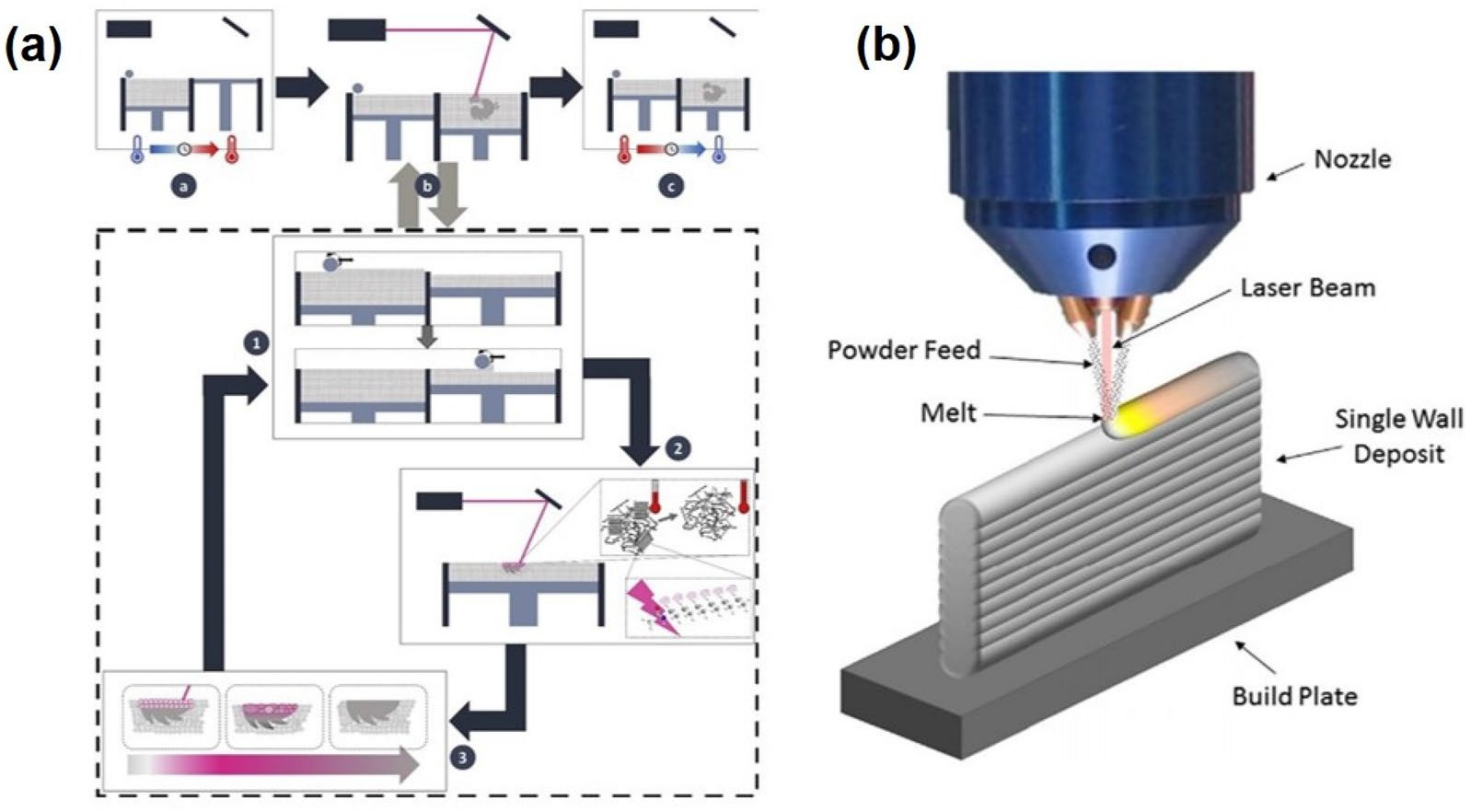
**a** Schematic illustration of powder bed fusion additive manufacturing process [59]. **b** Schematic illustration of directed energy deposition additive manufacturing process [53]

PBF and DED are two powerful and versatile AM techniques that are applicable to powder-based manufacturing systems in industry. Lasers are one of the most popular energy sources for the PBF and the DED techniques because the highly focused and spatially coherent laser beam irradiation enables efficient and area-selective melting or sintering of applied powder materials. The PBF and the DED can construct complex and novel 3D solid geometry in a layer-wise fashion, but their methodologies to build 3D structures are different. In the PBF method, a layer of powder is supplied onto a base plate and a focused laser beam translates onto the layer of the powder to selectively sinter or melt the powder along the path defined by a 3D CAD model. Then, the baseplate moves downward, and new layer of the powder is supplied onto the previously lasered powder bed. The 3D object is printed by repeating these steps, and unused powder can be collected and reused (Fig. 1a). Depending on whether the powder is sintered or completely melted, the PBF is often referred to as selective laser sintering (SLS) or selective laser melting (SLM), and a wide range of materials such as polymer, ceramic, or metals can be used for PBF. On the other hand, in the DED process, a nozzle head consisting of laser optics and a powder (or wire) delivery nozzle is mounted on a multi-axis arm (typically with 4 or 5 axes of freedom) which allows the nozzle head to freely move and deposit the materials from any angle (Fig. 1b). In comparison with the DED, the PBF can manufacture relatively complex geometry with high resolution, but printing size is rather limited.

Direct metal laser sintering (DMLS) is one of branded names for a metal SLM technique and was adapted by Ibrahim et al. to fabricate a 3D porous and scaffold structured stainless steel (SS) 316L electrode for metal/air cells [60]. The work aimed to develop 3D porous SS 316L electrode with large surface area and porosity to promote the electrochemical properties of SS 316L electrodes. They performed the DMLS process systematically and fabricated 25 cylindrical samples with varying laser power (30 W to 90 W) and scan speeds (300 mm/s to 1500 mm/s). The characterization results of the prepared SS 316L cylinders demonstrated that the porosity of the SS 316L cylinder increases with lower laser power and higher scan speed, while too low of laser power and too fast of a laser scan resulted in little to no sintering effect. The DMLS process with too high of a laser power and slow scan speed led to complete melt of the SS 316L powder and produced the highly dense SS 316L cylinders, which is not desirable for the use as battery cell electrodes. The authors suggested that laser parameters need to be optimized thoroughly for a preferable degree of partial powder melting that result in the desired porous structure of the SS 316L cylinder.

The SLS 3D printing was employed to fabricate highly porous graphite/polymer composite electrodes [61]. Mixtures of electronically conductive graphite powder and polymer supporting matrix such as polyamide-12, polystyrene, or polyurethane powder were used for the SLS process and the content of graphite powder in the powder mixture was varied from 5 to 40 wt.% to find out optimal composition that balances electronic and mechanical properties of the printed electrode. During the SLS process, a mechanically flexible solid structure containing homogeneously distributed accessible voids and graphite particles was built by sintering or partially melting the polymer supporting matrix powders. It was demonstrated that the graphite concentration, polymer matrix, porosity, and the SLS processing parameters are all very influential in the conductivity of the fabricated electrode. The author claimed that the SLS 3D printing technique enables fine-tuning of the mechanical parameters of the printed electrode by altering the selection of supporting polymer matrix, and the SLS process parameters and printed electrodes are suitable for use in redox flow cells.

Recently, developing a new Li-ion cathode with a rational 3D electrode microstructure has been one of the major challenges to improving specific energy and power capability of the Li-ion cells [62–64]. Schoenung and coworkers attempted to adopt the ceramic SLS technique using a laser engineered net shaping (LENS) system, which is one of the popular tools for the DED process [64] where a custom powder bed setup is equipped with the LENS system and performed a PBF-like process. Standard powder bed layer thickness of 100 μm was used for each laser beam scanning to fabricate 3D lithium nickel cobalt aluminum oxide (NCA) cathodes without any additives. A parametric single-track study was performed first to refine the SLS process parameters (volumetric laser energy density and laser beam diameter) and the optimum process parameters where the crack and the discontinuity were minimized were selected for 3D manufacturing of the NCA cathodes. Although electrochemical evaluation of the 3D printed NCA electrodes was not performed, this work revealed important information in regard to microstructure change and crystallographic transformations of NCA compounds present in the 3D printed NCA cathodes.

Based on our paper search, the PBF and the DED techniques have rarely been investigated for rechargeable cell components compared to other AM techniques in spite of their great advantages. However, the concept of the SLS (or SLM) process has been employed in various approaches, and those techniques are often referred to as direct laser writing (DLW). Iwabuchi et al. attempted to fabricate a thick and porous silicon-carbon nanofibers composite (Si-CNFs) anode for Li-ion cells using waste Si powder collected from Si wafer manufacturing [65]. The wet slurry composed of Si powder and CNFs dispersed in *N*-methylpyrrolidone (NMP) was prepared without a polymer binder and was cast onto a Cu current collector. Then, laser irradiation experiments were performed onto the cast film at various scan speeds to form a mechanically stable Si-CNF film by the SLS of the Si powders. The Si-CNF anode was not electrochemically evaluated in this paper, but the characterization results discovered two things: polycrystalline Si particles tend to be transformed to single crystal-like crystal structure after laser beam scanning, implying thermal annealing of the Si particles; a faster scan speed forms more micropores. More investigation on physicochemical properties and electrochemical behaviors of the Si-CNF electrodes are required to conclude optimum SLS process parameters for the Si-CNF electrodes, but this approach shows a new opportunity to manufacture binder-free Si anodes for Li-ion cells.

A two-step manufacturing process consisting of the extrusion 3D printing and the SLS techniques was developed by Yu et al. and used to fabricate a 3D aluminum (Al) anode for Al/air cells [66]. The 3D structure of the Al anode was built on an Al foil via the extrusion 3D printing process using highly viscous slurry composed of 75 wt.% of Al active nanoparticles and 25 wt.% of terpineol and ethyl cellulose mixture as a carrier. The high content of the organic carrier in the slurry was required for good printability in the extrusion process, but negative contribution of the electrically inactive component to the electrochemical behavior of the Al anode was concerned. To resolve the issue, the electronic conductivity of the 3D printed Al electrode was improved by the SLS process, which removes the organic carriers and sinters the Al nanoparticles in the electrode film through the photothermal method. Various laser powers from 3 to 20 W were tested and it was discovered 10 W was the optimal laser power because the film lasered at 10 W sufficiently removed the organic carrier while the Al nanoparticles were sintered well without evidence of serious ablation or agglomeration. The Al electrode fabricated at optimal laser power of 10 W showed the most promising electrochemical performance in the Al/air cell with 239 mAh/g discharge capacity at 0.95 V whereas a non-sintered anode sample exhibited only ~ 2 mAh/g.

In this section, the two representative LAM methods, the PBF and the DED techniques were briefly introduced, and the recent works relevant to those techniques were discussed. Although the abovementioned works are still at early-stage of research, they have shown their ability to fabricate 3D structured electrodes with conventional electrode materials such as graphite, Al, and NCA. There is no doubt that their ability for rapid manufacturing of complex 3D structures with little to no assistance of additional materials is unique, but substantial research effort is still required to successfully adopt these technologies to battery cell manufacturing processes.

## Direct laser writing for graphene electrodes

DLW is an emerging technique in battery research owing to its high fabrication preciseness, rapid production capability, scale-up potential, and incorporating flexibility with other manufacturing techniques, which enables making 3D features on a substrate precisely without the complicated requirements of chemical reagents or processing conditions (e.g., masking). DLW refers to various laser manufacturing techniques comprehensively, including LSM, LAM, or other selective photochemical device modifications. DLW has been a powerful tool for many electronic industries, and it has recently gained increasing attention from researchers for the applications that desire 3D patterns of conductive materials formed on various substrates.

Carbonaceous materials play an important role in both commercially available rechargeable cells and next-generation cells under development because of their excellent electrical conductivity, large surface area, low material cost, and good compatibility with other component materials. Recently, graphene, a single or a few layers of a 2D honeycomb lattice of carbon atoms, has been regarded as a promising electrode material for next-generation rechargeable cells because of its extraordinary electronic and mechanical properties along with its large surface area [67–70]. Studies on graphene anodes for Li-ion cells that reported dramatic improvement in specific capacity of the Li-ion cells have been performed [70], but the technical difficulty of handling the graphene powder due to its low material density and undesirable poor Coulombic efficiency of graphene anodes caused by its large surface area needs to be overcome for practical use.

**Fig. 2:**
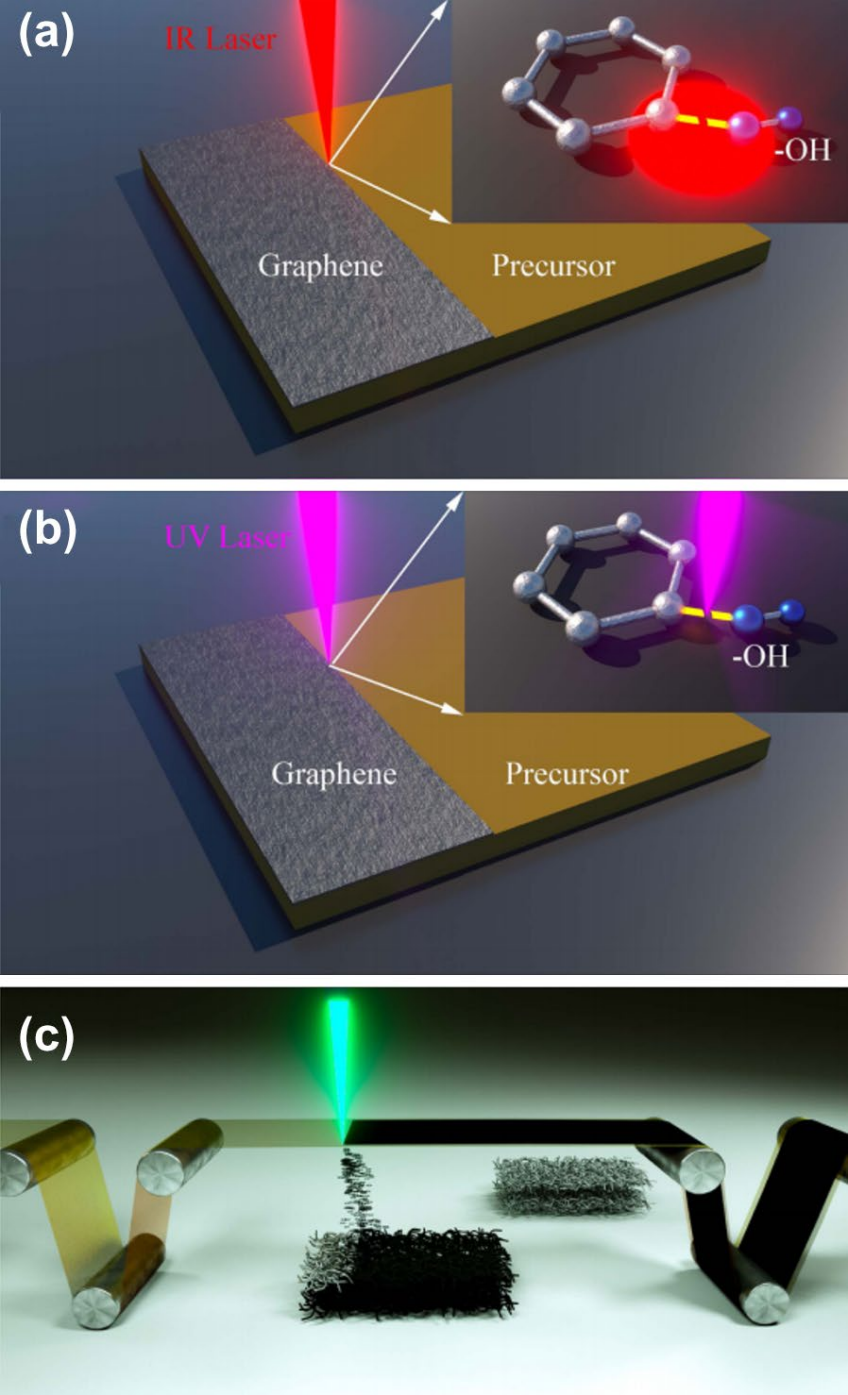
Schematic illustration of **a** the photothermal process within the laser writing of graphene electrode; the inset image demonstrates the thermal breaking between C and O–H bonds. **b** The photochemical process within the laser writing of graphene electrode; the inset image demonstrates the photon induced disassociation of band between C and O–H. **c** The laser induced forward transfer of graphene based on the roll-to-roll production with a polyimide film [76]

Despite the disadvantages of the graphene, it is still worthwhile using the graphene in the battery electrode to improve the electrochemical reaction rate, and the DLW technique has been investigated to open up new opportunities for the efficient processing of graphene [71–75]. There are two approaches to fabricate graphene electrodes using a laser: laser reduced graphene (LRG) prepared by laser thermal reduction of graphene oxide [72]; laser induced graphene (LIG) directly graphitized from raw materials such as polyimide (PI), polyetheretherketon (PEEK), and carbohydrates via laser photothermal conversion of sp3-carbon atoms to sp2-carbon atoms [71, 73]. The 3D structured LIG can simply be fabricated by a one-step graphitization process of the polymeric precursors and the entire polymer film is often converted to the highly porous graphene (Fig. 2), but the selective graphitization of the polymer layer can also be performed to create graphene patterns on the electrode. The LRG method tends to be more complicated than the LIG method because it requires a multiple-step process consisting of graphene oxide synthesis, graphene oxide film coating, and reduction, but either fabrication techniques can be chosen depending on the substrate material, the fabricate condition, or chemical compatibility [76].

Recently, many interesting studies adopted the DLW to fabricate the 3D structured graphene electrodes for various rechargeable cells [70, 74–80]. Ren et al. reported a bifunction Co_3_O_4_/LIG catalyst for improved oxygen reduction reaction (ORR) and oxygen evolution reaction (OER) in metal-air batteries [78, 80]. In the paper [80], the Co_3_O_4_/LIG catalytic electrode was prepared by the DLW method, and a brief description of the fabrication procedure is as follows: a Co(NO_3_)_2_ precursor solution was dropped into the LIG film on the PI substrate fabricated by the DLW technique and lased again under the same laser operation conditions to convert Co(NO_3_)_2_ to Co_3_O_4_. Because graphene [81] and first-row transition metal oxides are known to be promising alternative materials to expensive noble metal catalysts, the authors aimed to develop noble metal-free bifunctional electrocatalysts by incorporating both materials in a catalytic electrode using the DLW method. The Co_3_O_4_/LIG composite catalyst showed a high-power density of 84.2 mW/cm^2^ at 100 mA/cm^2^ in the Zn-air cell, which is attributed to their good electrocatalytic activity. Ren et al. developed another promising bifunctional OER/ORR catalyst consisting of MnNiFe/LIG using the DLW technique [78]. The promising performances of MnNiFe/LIG catalytic electrode with areal capacity of 26.3 mAh/cm^2^ at 2.0 V and reversible cycling performance of > 100 cycles with a cutoff capacity of 0.4 mAh/cm^2^ was demonstrated in Li-O_2_ cells without the presence of a redox mediator [78].

Zhang et al. fabricated a binder-free 3D LIG film anode adhered on the Cu foil using the DLW technique performed at N_2_ atmosphere. The DLW process at N_2_ atmosphere leads to nitrogen doping of 3D LIG film that improves the surface wettability and electronic conductivity of the LIG film [77]. The prepared nitrogen-doped LIG electrodes were tested as the anode for a Na ion cell showed promising electrochemical performance with a high specific capacity of 425 mAh/g at 0.1 A/g while most of the carbon-based anodes exhibit ~ 400 mAh/g or below. Rate of capability and cycle stability of the nitrogen-doped LIG electrode was also improved which are attributed to the improved electrolyte permeability and electronic conductivity of the electrode, and the best cell performance was obtained when the PI film was completely converted into the LIG.

The DLW method also showed effective electrode modification for a Li metal anode for high energy Li metal rechargeable cells. The biggest challenge of the Li metal anodes is an uncontrollable formation and growth of dendritic Li that can short-circuit the cell, causing cell death or possibly leading to a fire [82–84]. Yi et al. reported an interesting study on the stabilization of the Li metal anode by regulating the Li nucleation and deposition kinetics with the LIG [85]. They designed a 3D-hierarchical composite electrode composed of a copper current collector, a pillared array of flexible PI, and the porous LIG on the walls of the PI pillars; namely, LIGHS@Cu was prepared by the DLW process. High Coulombic efficiency of ≈ 99% at 1 mA/cm^2^ with a long cycle life of over 400 h (corresponding to 200 galvanostatic cycles) was achieved in the Li/LIGHS@Cu cells whereas the Li/bare Cu cell was dead after about 50 cycles. The authors claimed that the improved stability is mainly attributed to the high number of defects and heteroatoms in the LIG that significantly lowers the Li nucleation barrier compared to the conventional Cu foil. Another approach to stabilize the Li anode was reported by Chen et al., who developed a laser-induced silicon oxide (Li-SiO_x_) layer which was derived from a silicone-based adhesive material of commercial PI adhesive tape [86]. It was demonstrated that the Li-SiO_x_ film on the Cu current collector successfully suppressed the formation of dendrites, and delivered 99.3% higher Coulombic efficiency compared to bare electrodes, and improved cycle life by three times in the Li-SiO_x_-Cu/LiFePO_4_ cells relative to the uncoated Cu-LiFePO_4_ cells.

The lithium/sulfur (Li/S) cell is another emerging Li rechargeable cell system for high specific energy applications due to its outstanding theoretical specific energy of 2600 Wh/kg, which is about 4–5 times larger than conventional Li-ion cells [8, 87, 88]. There are several technical challenges that prevent commercialization of Li/S cells and polysulfide (Li-PS, Li_2_S_x_, x = 4–8) shuttling effect is one of the major challenges to overcome. Li-PS that is highly soluble in most liquid electrolyte forms and diffuses out from the S electrode during the cell cycling and the Li-PS diffused to the Li electrode is deposited onto the Li metal surface as Li_2_S, which causes substantial degradation of durability and reliability of the Li/S cells. Alhajji et al. developed a freestanding LIG interlayer using the DLW method and the LIG interlayer was placed between the Li metal anode and S cathode to suppress diffusion of Li-PS towards the Li metal anode [79]. The Li/S cell with the LIG interlayer exhibited a high specific capacity of 1160 mAh/g with stable capacity retention (80.4%) over 100 cycles while majority of other works report capacity retention less than 80% after 100 cycles.

In summary, the DLW techniques have widely been employed for the fabrication of various graphene-based battery cell components such as active electrodes, catalysts, interlayers, and functional coating layers. The simple and cost-effective DLW process has shown great potential to create functional graphene 3D structure and the rechargeable cells with the 3D LIG exhibited significant improvement in cell performance. Most works focused on the conversion of precursor layers into graphene; however, there is a lack of thorough investigation on chemical and crystallographic structures and morphology of the synthesized graphene, which are also very influential in electrochemical behavior of the rechargeable cells.

## Laser-induced forward transfer for rechargeable cells

Inkjet printing is one of the most extensively studied droplet-based direct ink writing techniques to fabricate 3D printed electronic devices. The technique enables the printing of electronic materials with ~ 20 μm resolution and the micropatterns are easily programmable [89]. Compared to other vacuum-based deposition methods (e.g., photolithography or chemical vapor deposition), the inkjet printing has substantial advantages in the cost-efficiency, scalability, and versatility. In recent years, some functional materials were adopted as an ink material for the inkjet printing (e.g., graphene), imparting additional functionality to the printed devices [90]. However, the ink material is often limited by its rheological properties, excluding several promising functional materials, e.g., carbon nanotubes or nanowires [91].

**Fig. 3:**
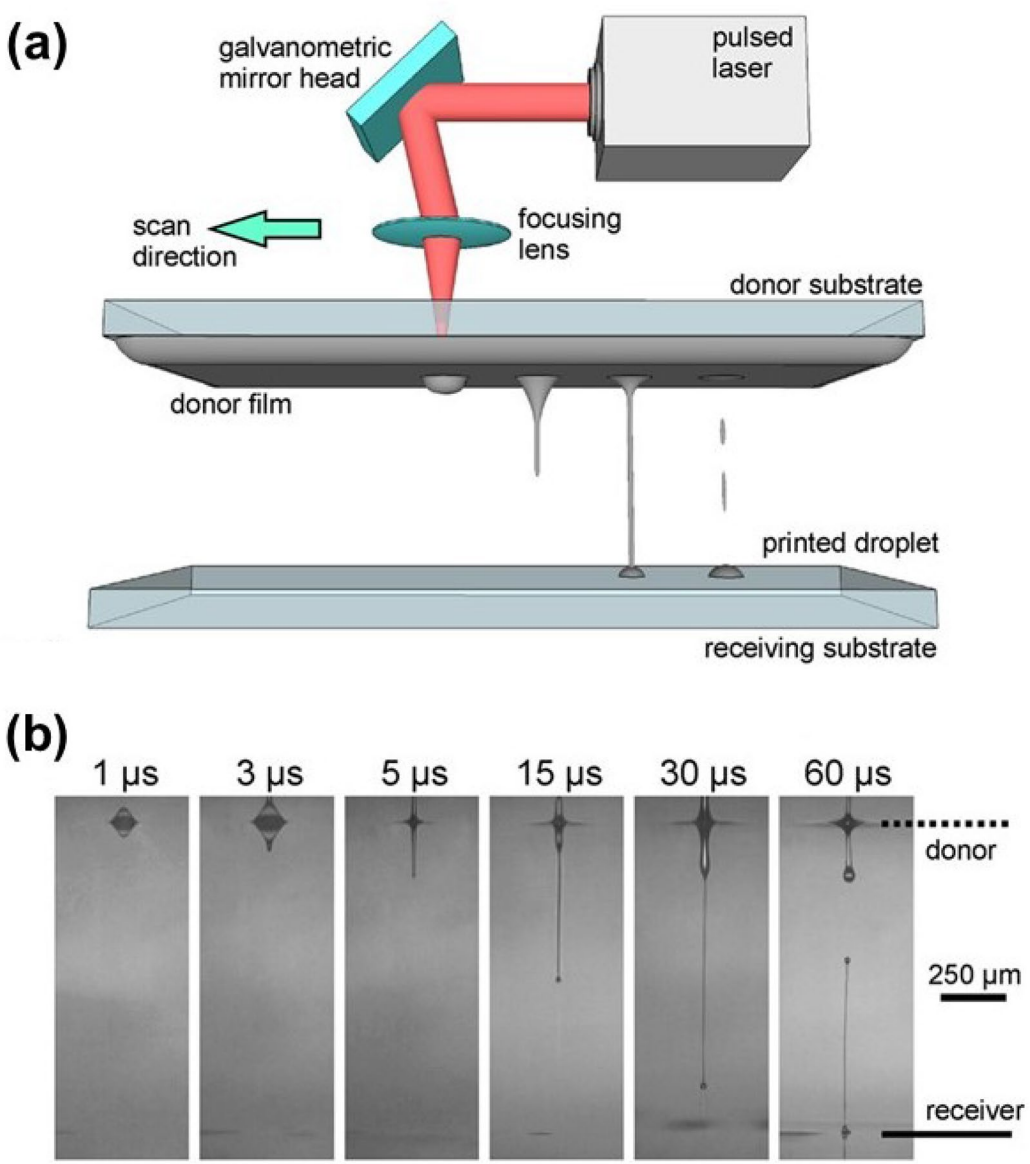
**a** Schematic illustration of the LIFT of liquid films. The pulsed laser beam is translated along the scan direction and a liquid jet is formed upon laser irradiation which transfer the donor material to the receiving substrate. **b** Stop action movie of the LIFT process show the jetting dynamics characteristic [91]

LIFT is a direct printing technique using a sacrificial donor film (Fig. 3). The LIFT reserves the advantages of the inkjet printing technique while the transferring materials are less limited by the rheological properties of the ink. Thus, the forementioned functional materials can readily be printed on various substrates (solid or flexible support) without a rheological consideration. LIFT typically utilizes a pulsed laser beam which is focused on a thin film of the ink (donor film) to subsequently form a liquid jet and then an ink droplet propagates away from the donor film. A receiving substrate is placed facing the donor film and the ink is directly transferred to the surface of the receiving substrate, collecting the ejected droplet (Fig. 3). In contrast to DLW, LIFT does not involve chemical modification of the transferred material. The fundamentals and applications of LIFT is well reviewed elsewhere [58]. Here, we limit our scope to the applications of LIFT to energy storage devices, especially rechargeable batteries.

Several works have been reported on LIFT to transfer electrode materials on a metal-based current collector surface to generate microbatteries. Wartena and coworkers used LIFT to print composite powders of electrode materials, i.e., LiCoO_2_ mixed with carbon and binders for the Li-ion cathode, and a composite of carbon and binder for the Li-ion anode, onto laser-micromachined metal-foil current collectors (Cu and Al) [92, 93]. It was revealed that the LiCoO_2_ transferred by LIFT exhibits the preferred crystallographic orientation of the particles in the (003) direction relative to non-laser transferred materials. While the laser energy does not alter the preferred orientation, the number of passes and transfer distance both have a significant influence on the texture. [94] The fabricated half cells and packaged microbatteries demonstrated capacities of ~ 100 mAh/g, which are comparable to the capacities of stenciled and pressed electrodes. The electrodes prepared by LIFT are not sintered and thus the volumetric capacity of the electrodes is lower than those reported for sputtered thin film microbatteries. One strategy to overcome this is to make the electrodes thicker to deliver the same amount of charge as the sputtered and sintered electrodes. Indeed, Kim et al. laser-printed a thick-film LiCoO_2_ and carbon electrodes, and the electrodes are ionically connected through gel polymer electrolytes. The laser printed microbatteries exhibited an order of magnitude higher areal capacity of ~ 2586 mAh/cm^2^ than that reported for the sputter-deposited thin-film microbatteries (~ 160 mAh/cm^2^) [95]. Arnold et al. demonstrated the production of planar alkaline microbattery cells by LIFT printing Zn anodes and Ag_2_O cathodes in planar configurations and used laser micromachining to ultimately shape the deposited materials into the microbattery cells [96]. The alkaline microbattery cells exhibited high areal capacity of 270–450 mAh/cm^2^ which is greater than Li microbatteries (~ 155 mAh/cm^2^) [97] and comparable with the nickel-zinc alkaline microbatteries (389 mAh/cm^2^) [98].

A notable advantage of LIFT that has not yet been discussed is that the active materials can be transferred without employing organic solvents. Rosenberg et al. reported the LIFT transfer of MnO_2_, which were prepared to be used as a catalyst for Na/air cells [99]. Instead of using organic solvents, a photosensitive polymer, i.e., triazene polymers (TP), was used as a dynamic releasing layer (DRL). Upon a UV laser irradiation, the photosensitive TP decomposes into gaseous fragments, releasing the active material to be transferred to the current collector substrate. The electrochemical characterization of sodium-air cell exhibits a high specific charge (~ 2000 mAh/g) and high retention rate (85% after 55 cycles) compared to cells prepared by traditional wet methods. Constantinescu et al. pre-synthesized 2D-arrays of carbon nanowalls (CNW) and subsequently transferred the as grown CNWs onto a flexible polyimide sheet without solvent [100]. Raman spectra revealed that the vibrational band structure was preserved after the LIFT printing, indicating that the high structural fidelity of the printed materials to the as-grown donor film. The result promises LIFT can be an effective technique to prepare electrode materials on a flexible substrate that can potentially be adopted in flexible batteries. Li et al. additively deposited synthesized graphene flakes onto nickel foam using the LIFT technique [101]. Then the deposited graphene layer was post-annealed by laser scribing to enhance the lattice matching between the extended basal plane of graphene and Ni (111). The LIFT transferred and post-treated electrode demonstrated high electrical conductivity, high retention rate and large areal specific capacitance, and power density.

Despite its great simplicity compared to other deposition techniques, there are efforts to optimize the LIFT process to enhance cost-effectiveness and versatility. Sopena and coworkers used continuous wave (CW-LIFT) instead of pulsed lasers, which allows a reduction in the cost of the printing system [91]. While the pulsed wave LIFT prints rasterized pixels, the CW-LIFT enables printing of continuous line and printing is well optimized at lower laser power (1 W) than conventionally used regime. They printed Ag nanoparticles and carbon nanofibers (CNF) on various substrates (i.e., glass, polyimide, and paper) and demonstrated that the conductive lines can be printed in continuously.

In summary, LIFT is a powerful transfer technique to print functional materials on various substrates without many limitations on the choice of ink materials or substrates. LIFT can deposit active electrode materials on solid or flexible substrates without chemical modification and lithium or alkaline microbatteries can be successfully fabricated. The cost-effectiveness and nozzle-free features make LIFT a viable technique for micropatterning. However, challenges are present, e.g., process modeling and optimization, and long-term stability of LIFT-printed objects that need to overcome to make this technology more prevalent.

## Laser ablation subtractive manufacturing

LSM is a type of manufacturing that shapes an object in 3D via material removal processes using a focused laser beam and the laser beam toolpath is generally driven by computer numerical control (CNC). Similar to LAM, LSM has been very popular to fabricate prototypes or end-use parts that require tight tolerances and geometries that are not easy to be manufactured by traditional casting and molding methods. Although LAM offers the better capability to construct more complex shapes and hollow internal structures, LSM is generally more flexible in the selection of materials and size of objects they can work with and can achieve a better finishing than LAM without post-processing. As opposed to LAM, LSM creates microfeatures of materials by removing the material in local areas where a focused laser beam is irradiated. During the LSM process, a focused laser beam penetrated an object’s surface and generates heat upon the interaction of high-power radiation with materials, resulting in vaporization of the materials [102]. The vaporization process is often referred to by a few different terms such as laser ablation, laser vaporization, laser etching, and laser sputtering, and we will refer to the term ‘laser ablation’ in this paper. A pulsed laser that turns on and off extremely quickly, in the order of up to femtoseconds, is widely used for laser ablation techniques because it can ablate the materials within the region of interest with almost negligible thermal damage to the surround area. Lasers operated at different wavelengths should be used as appropriate for the material being irradiated and the processing parameters such as laser power, pulse time, and translation speed are adjusted to optimize the final product. The laser ablation technique is often used to cut the workpiece or to create linear patterns on the objects, but it can also create a 3D structure using multiple beams or a layered approach to build the structure.

One of the most popular device processing technologies that uses the laser ablation method is the pulsed laser deposition (PLD) technique. PLD uses a laser aimed at a target material to evaporate it into a plasma plume, which rises and deposits on the substrate to form a thin film. PLD has been intensively investigated for a wide range of applications and there are many scientific research articles reported about the PLD technique to fabricate thin film battery electrodes. Since this review mainly focuses on the 3D manufacturing approaches to design more complex microstructures of the battery cell components, we will not review the PLD technique, but there are good review articles written by other researchers [103–108].

Besides PLD, the laser ablation method has been used for cutting conventionally fabricated electrode sheets into a desired size or shape [109–112]. In the battery cell manufacturing process, the fabricated electrodes are mechanically cut to size using a die cutter and stacked with other cell components. One issue associated with the mechanical cutting process of the electrodes is the delamination of the electrode film or the creation of burr on the cutting edge, which can cause an undesired degradation of the electrochemical durability of the rechargeable cell [110]. Significant effort for the cutting tool’s maintenance is often required for reliable electrode manufacturing because the die cutter can eventually warp out of shape. In contrast to the conventional mechanical cutting process, laser ablation cutting can result in a much smoother and cleaner cutting edge of the electrodes, so the negative impact caused by the electrode cutting process can be minimized. Excellent design flexibility and rapid processing time of the laser ablation cutting are also great benefits for the electrode manufacturing [109–112]. Lutey et al. investigated the laser cutting method on Li iron phosphate (LiFePO_4_, LFP) cathodes and graphite anodes for the Li-ion cells with varying over 12 discrete parameter groups in order to optimize the laser cutting process parameters [110]. Their results demonstrate that the pulsed lasers were preferable over continuous wave lasers because the continuous wave laser exhibits less ablation depth with larger thermal damage to the LFP film. Because the light absorption efficiency of materials largely depends on the physical properties of the materials, the laser ablation process for multi-component layers like battery electrodes consisting of an electrode film and a metallic current collector could be complicated. The results in the paper suggest that the laser parameters should be optimized with respect to metallic current collectors for high cutting efficiency and good quality of the electrode cutting edge.

**Fig. 4:**
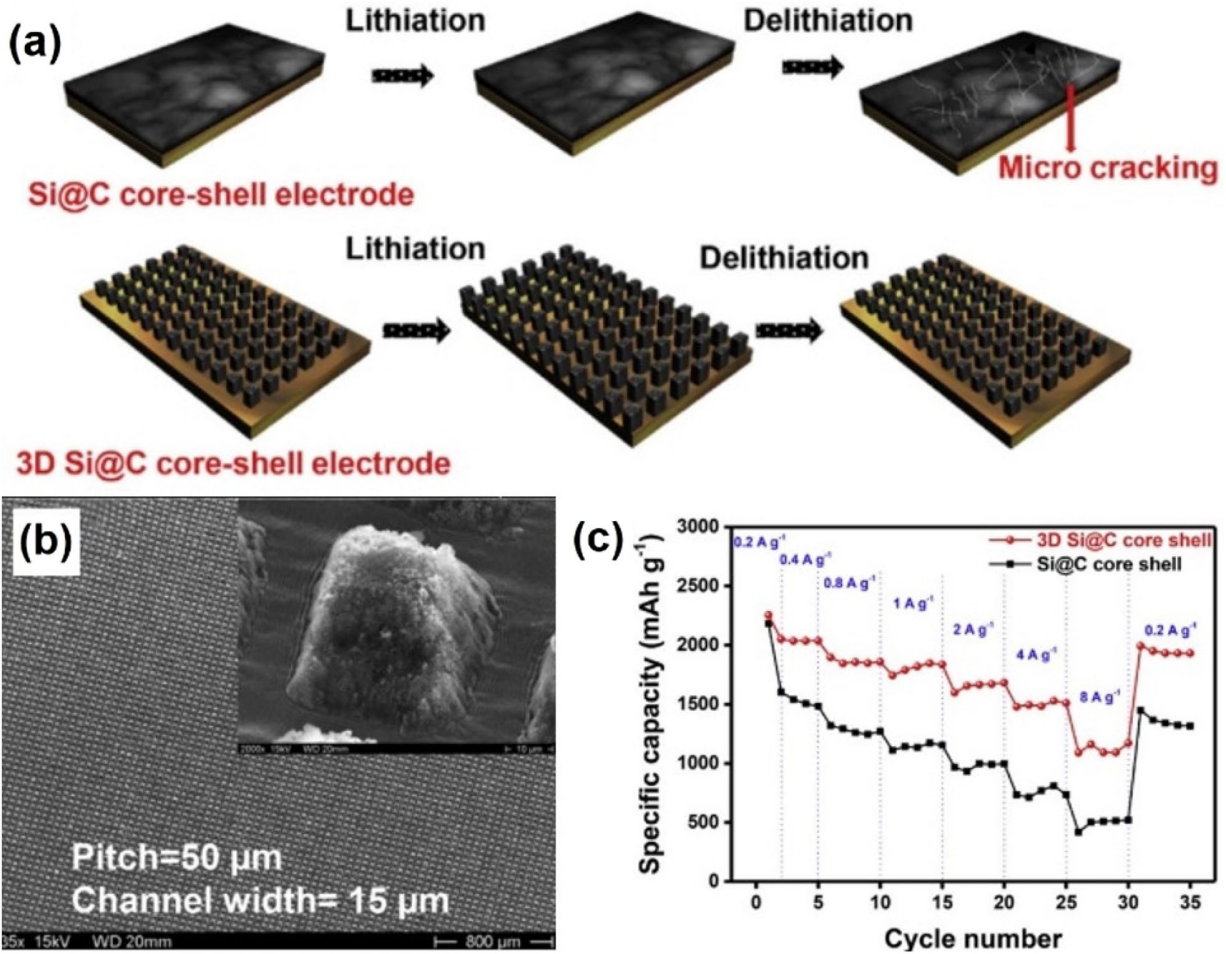
**a** Schematic illustration of the 3D Si@C core–shell electrode. **b** SEM images of the 3D Si@C core–shell electrodes. **c** Rate performances of the Si@C and 3D Si@C core–shell cells at various current densities of 0.2, 0.4, 0.8, 1, 2, 4, 8, and 0.2 A/g (cut-off voltage: 0–2 V) [128]

Although the laser cutting process showed promising opportunity to advancing the electrode manufacturing process, laser electrode cutting does not fully utilize the excellent capability of the laser ablation method in precision and selective manufacturing. The surface morphology modification of the electrode via the laser ablation technique is an interesting approach in this regard because it can create a low tortuous pore network at the surface of the electrode film, which not only enlarges the surface area of the electrode but also improves the electrolyte permeability and ion transport kinetics through the electrodes [113–118]. Kohler and his coworkers reported a series of experiments [119–128] which investigated laser-assisted microstructures for Li-ion cell electrodes. In one of their reports, a carbon coated silicon core–shell (Si@C) electrode was fabricated using the conventional tape casting method and the 3D structure was created by the laser ablation technique [128]. To mitigate the mechanical pulverization issues of Si electrode caused by a huge volume change of Si particles during the cycling [129], the 3D ablation technique is performed on Si@C electrodes to form a laser-ablated pattern that accommodates the volume change of the Si particles (Fig. 4a). The 3D Si@C electrodes were successfully fabricated with optimized laser ablation processing parameters and the morphology of the 3D Si@C electrode is shown in the SEM image (Fig. 4b). The electrochemical evaluation results verify the dramatic improvement of the 3D patterned Si@C electrode in rate performance at all tested current densities (Fig. 4c) and structural stability of the electrodes during cycling compared to the unstructured Si@C electrode. The improved high rate capability of the 3D Si@C electrode is attributed to the 3D pattern of the Si@C electrode which not only promotes fast Li ions transport through the Si@C electrode, but also suppresses mechanical fracture of the electrode by accommodating the volume change of the Si particles.

While the 3D laser ablation techniques have also been investigated for metal oxide Li ion cathodes, it was found that the laser ablation process can result in annealing of the metal oxide active materials [119, 122, 130–132]. In the previous work performed by Kohler et al., a LiCoO_2_ (LCO) thin film was deposited onto a stainless-steel substrate by R.F magnetron sputtering and the surface of the LCO thin film was modified by pulsed laser irradiation [119]. While the cone-shaped periodic surface structures that increases surface area of the LCO cathode were produced as a result of the laser irradiation onto the LCO thin film cathode, the crystal structure of the LCO film also changed from rock salt structure to the electrochemically favorable layer-structure. The electrochemical behavior of the LCO thin film electrode was dramatically improved with approximately 1.7 times higher specific capacity than that of the as-prepared LCO thin film electrode at C/4. The results also discovered that the higher annealing degree of the LCO film can be obtained at higher laser energy density though too high of a laser energy density can destroy the cone-shaped electrode surface. Therefore, it was suggested that laser energy densities need to be adjusted thoroughly to create 3D surface structure with desired crystal structure [119].

Additional investigations were performed at different annealing temperatures in heat-affected-zone by adjusting laser power of up to 50 W [131, 132]. The temperature in the range of 200–700 °C was measured in the heat-affected-zone using a pyrometer in-situ during the laser ablation process, and the electrode that was laser-annealed at 400 °C showed the most enhanced specific capacity which is about 4–6 times higher than that of the unstructured electrode at C/1.25 over 350 cycles. The authors emphasized that the laser annealing process takes only about 10 min while the conventional furnace annealing generally requires a long processing time of 20–60 h, which is a great benefit for time- and energy-efficient electrode manufacturing.

A similar laser ablation structuring concept was implemented by Prὅll and his coworkers for a Li nickel manganese cobalt oxide (Li(NiMnCo)O_2_, NMC) cathode that is regarded as a cost-effective high-energy-density cathode for the Li-ion cells [130, 133]. In the paper [133], they fabricated the thick NMC cathode using a conventional tape casting method and then created 3D architectures using the laser ablation method. The laser 3D structured NMC electrode showed approximately 10% higher specific capacity that the conventionally fabricated NMC electrode in a pouch cell over 1000 cycles, which is due to better electrolyte permeability of the laser structured NMC electrode than the unstructured NMC electrode. A similar investigation on laser-engineered thick NMC cathodes was performed by other researchers [117] with different electrode thicknesses and porosities, from 100 to 210 µm and 26% and 50%, respectively. In the similar trend as observed for the Prὅll’s work [133], where the laser-structured NMC electrode shows the improved cell performance, it should be noted that the capacity differences between the laser-structured NMC electrode and the unstructured NMC electrode became more significant at higher test rates. These results were supported by improved Li-ion diffusivity and lower cell polarization of the laser-structure thick NCM electrode measured experimentally, which is mainly attributed to the uniformly aligned 3D micro-groove structure of the laser-structured NMC electrode.

One other way to use the laser ablation technique is to create a dotted pattern across the surface of electrodes [113–115]. The dotted patterns on the electrodes work the same way as the pore channels created by the linear translation of the focused laser beam do, but the dotted laser scanning can be more cost-efficient since it may remove less amount of materials than linear laser scanning. In the paper reported by Habedank et al. [114], the dotted patterns were created by the laser ablation onto a graphite anode and the laser-structured graphite anodes were found to deliver 20% higher discharge capacity at 2C or higher rates compared to the unstructured graphite anode. The authors also discovered high laser peak fluence with a single laser scanning to be more effective at ablating the graphite anode as repetitively using high pulses could damage the anode and degrade cell performance [114]. This work was followed by another experiment that studies the effect of laser-structuring on the electrolyte permeability of the graphite anodes [115]. Using in situ neutron radiography, they proved that the laser-structured graphite anode with low porosity (30%) in a pouch cell could be wet up to 100% in about 15 min, while the unstructured graphite anode with the same porosity, as well as the graphite anode with even higher porosity of 40%, took several hours to wet. This finding emphasizes that the laser ablation technique can reduce the production costs of the Li-ion cells by shortening the time for cell manufacturing. Kim et al. also illustrated a possible new concept of the roll-to-roll electrode fabrication process that integrates a laser ablation step after the calendaring process [116]. They claim that the technical barrier to integrate the laser ablation in the roll-to-roll electrode fabrication process is not high, since the laser ablation technique has been already used in wide range of industries and it would not need any additional materials for the process. In the paper, they performed galvanostatic polarization interrupt test for the laser structured graphite anodes and showed that the pore channels with low tortuosity created by the laser ablation technique can promote transport kinetic of Li-ions.

In summary, various approaches that use laser ablation techniques to engineer battery electrodes were discussed. The laser ablation techniques have proved their ability to create 3D microstructure while suppressing damages to the surroundings. The improved cell performance was demonstrated for various electrodes for Li-ion cells and especially the high-rate performance of the laser-structured electrodes is very impressive. It was suggested that the improved high-rate performance of the laser-structured electrodes was attributed to high surface area and low tortuosity and improved electrolyte permeability of the laser-structured electrodes. Overall, the laser ablation techniques have a lower technical barrier to be integrated in state-of-the-art battery electrode manufacturing lines than LAM since conventional battery electrodes can be easily processed by the laser ablation technique according to the above-discussed papers. Nevertheless, the laser ablation techniques have the potential drawback that needs to be addressed, that is, the increase of battery cell manufacturing cost ($/kWh). Considering the fact that the cost contribution of the battery electrode materials is very high (up to about 40% of the total cell manufacturing costs), constructing the 3D structure of the electrode by the removal of the electrode materials is not cost efficient. The degree of the material ablation may need to be limited to some degree where the production cost is not increased by the laser ablation process, while improving the cell performance. Therefore, a more systematic research and development plan is desired to minimize the impact on the production cost of rechargeable cells.

## Summary and outlook

This paper provides a comprehensive review of various laser 3D manufacturing technologies and the techniques that are introduced and categorized into four different sections: selective laser sintering techniques; direct laser writing techniques for graphene-based electrodes; laser-induced forward transfer techniques; laser ablation subtractive techniques. The concepts and respective advantages of each laser manufacturing technique are briefly discussed and notable studies that address current and emerging issues of rechargeable cell manufacturing and their achievement are introduced and emphasized. Several works have accomplished dramatic improvement in electrochemical performance of the rechargeable cells, but it is worthwhile noting that those approaches are still at an early development stage and many technical challenges need to be overcome in order to be competitive with the conventional manufacturing process.

One of the biggest concerns of laser manufacturing technologies is the selection of an appropriate laser for specific printing materials because the laser manufacturing efficiency and spatial resolution of printing structure strongly depend on the laser properties and laser-material interaction. Configuration of the laser manufacturing equipment likely needs modifications depending on printing materials unlike the conventional roll-to-roll manufacturing equipment which is relatively flexible in changing materials. This issue raises another question about how efficient the laser manufacturing techniques will be for multi-component systems. Conventional rechargeable cell electrodes are generally composed of 2–4 different solid constituents such as active material, conductive carbon, and a polymer binder. Even if the laser manufacturing techniques enable fabrication of a binder-free electrode via selective melting or sintering process, the electrodes may still need electronic conductive additives in them. So far, previous works have mainly been used for a single constituent system and their physicochemical and electrical properties have not been sufficiently demonstrated yet. In addition, the construction of rational micrometer-scale 3D structure has been a major goal of laser manufacturing techniques and relatively little attention has been given to nano-scale pores that is also very influential in the performance of rechargeable cells. Large scale and continuous manufacturing capability while addressing abovementioned issues will also be a technical barrier that prevents commercialization of the laser manufacturing technologies, thus enormous research effort still needs to be dedicated to investigating laser 3D manufacturing techniques.

Some important practical terms for commercial batteries such as volumetric energy or power density, and material cost can be other issues for 3D design and manufacturing technologies, especially for those which create a 3D structure by ‘removing’ materials. Systematic computational and experimental design strategies will be highly desired to minimize the loss of materials and excess internal pore volume of battery electrodes so that 3D manufactured batteries can become competitive to the conventional batteries. Despite the several challenges, there is no doubt that laser manufacturing technologies have their unique strengths and amazing potential to become a game changer of the rechargeable cell manufacturing. The great 3D structure construction capability of the laser manufacturing will enable improvements to specific energy and power density of rechargeable cells, and they can possibly lower manufacturing cost by reducing material wastes or simplifying manufacturing processes. Hybrid laser manufacturing technologies consisting of a combination laser manufacturing and conventional manufacturing or other advance manufacturing techniques are also considerable to open new opportunities for advanced manufacturing capability that has not been demonstrated by any conventional methods. A fully 3D printed rechargeable batteries is an ultimate goal of all advanced 3D manufacturing and we believe that laser manufacturing will play a key role to realize it.

## Data Availability

Not applicable.

## References

[1] Nagaura T (1990). Lithium ion rechargeable battery. Progr. Batter. Solar Cells.

[2] Chayambuka K, Mulder G, Danilov DL, Notten PHL (2018). Sodium-ion battery materials and electrochemical properties reviewed. Adv. Energy Mater..

[3] Fang G, Zhou J, Pan A, Liang S (2018). Recent advances in aqueous zinc-ion batteries. ACS Energy Lett..

[4] Gummow RJ, Vamvounis G, Kannan MB, He Y (2018). Calcium-ion batteries: current state-of-the-art and future perspectives. Adv. Mater..

[5] Lau J, DeBlock RH, Butts DM, Ashby DS, Choi CS, Dunn BS (2018). Sulfide solid electrolytes for lithium battery applications. Adv. Energy Mater..

[6] Mauger A, Julien CM, Paolella A, Armand M, Zaghib K (2018). A comprehensive review of lithium salts and beyond for rechargeable batteries: progress and perspectives. Mater. Sci. Eng. R. Rep..

[7] Abakumov AM, Fedotov SS, Antipov EV, Tarascon J-M (2020). Solid state chemistry for developing better metal-ion batteries. Nat. Commun..

[8] Hwa Y, Cairns EJ (2020). Nanostructured sulfur and sulfides for advanced lithium/sulfur cells. ChemElectroChem.

[9] Um JH, Kim K, Park J, Sung Y-E, Yu S-H (2020). Revisiting the strategies for stabilizing lithium metal anodes. J. Mater. Chem. A.

[10] Guo Q, Zeng W, Liu S-L, Li Y-Q, Xu J-Y, Wang J-X, Wang Y (2021). Recent developments on anode materials for magnesium-ion batteries: a review. Rare Met..

[11] Zhang C, Huang K (2021). A Comprehensive review on the development of solid-state metal–air batteries operated on oxide-ion chemistry. Adv. Energy Mater..

[12] Walsh FC (1992). The Kinetics of electrode reactions: part II—mass transfer and mixed control. Trans. IMF.

[13] Hawley WB, Li J (2019). Electrode manufacturing for lithium-ion batteries—Analysis of current and next generation processing. J. Energy Storage.

[14] Kim KM, Jeon WS, Chung IJ, Chang SH (1999). Effect of mixing sequences on the electrode characteristics of lithium-ion rechargeable batteries. J. Power Sources.

[15] Paredes JI, Burghard M (2004). Dispersions of individual single-walled carbon nanotubes of high length. Langmuir.

[16] Liu G, Zheng H, Song X, Battaglia VS (2012). Particles and polymer binder interaction: a controlling factor in lithium-ion electrode performance. J. Electrochem. Soc..

[17] Kraytsberg A, Ein-Eli Y (2016). Conveying advanced li-ion battery materials into practice the impact of electrode slurry preparation skills. Adv. Energy Mater..

[18] Lim S, Ahn KH, Yamamura M (2013). Latex migration in battery slurries during drying. Langmuir.

[19] Baunach M, Jaiser S, Schmelzle S, Nirschl H, Scharfer P, Schabel W (2016). Delamination behavior of lithium-ion battery anodes: influence of drying temperature during electrode processing. Dry. Technol..

[20] Jaiser S, Müller M, Baunach M, Bauer W, Scharfer P, Schabel W (2016). Investigation of film solidification and binder migration during drying of Li-Ion battery anodes. J. Power Sources.

[21] Font F, Protas B, Richardson G, Foster JM (2018). Binder migration during drying of lithium-ion battery electrodes: modelling and comparison to experiment. J. Power Sources.

[22] Guerin K, Fevrier-Bouvier A, Flandrois S, Simon B, Biensan P (2000). On the irreversible capacities of disordered carbons in lithium-ion rechargeable batteries. Electrochim. Acta.

[23] Shim J, Striebel KA (2003). Effect of electrode density on cycle performance and irreversible capacity loss for natural graphite anode in lithium-ion batteries. J. Power Sources.

[24] Chung D-W, Ebner M, Ely DR, Wood V, Edwin García R (2013). Validity of the Bruggeman relation for porous electrodes. Model. Simul. Mater. Sci. Eng..

[25] Li J, Christensen L, Obrovac MN, Hewitt KC, Dahn JR (2008). Effect of heat treatment on Si electrodes using polyvinylidene fluoride binder. J. Electrochem. Soc..

[26] Jeong G, Lee SM, Choi NS, Kim Y-U, Lee CK (2011). Stabilizing dimensional changes in Si-based composite electrodes by controlling the electrode porosity: An in situ electrochemical dilatometric study. Electrochim. Acta.

[27] Hwang SS, Sohn M, Park H-I, Choi J-M, Cho CG, Kim H (2016). Effect of the heat treatment on the dimensional stability of Si electrodes with PVDF Binder. Electrochim. Acta.

[28] Sakamoto JS, Dunn B (2002). Hierarchical battery electrodes based on inverted opal structures. J. Mater. Chem..

[29] Deville S (2008). Freeze-casting of porous ceramics: a review of current achievements and issues. Adv. Eng. Mater..

[30] S. Behr, Freeze casting of NCA scaffolds for application in lithium-ion batteries, Diploma Thesis, (2012).

[31] Vu A, Qian Y, Stein A (2012). Porous electrode materials for lithium-ion batteries—how to prepare them and what makes them special. Adv. Energy Mater..

[32] Singh DP, Mulder FM, Wagemaker M (2013). Templated spinel Li_4_Ti_5_O_12_ Li-ion battery electrodes combining high rates with high energy density. Electrochem. Commun..

[33] Hedayat N, Du Y, Ilkhani H (2017). Review on fabrication techniques for porous electrodes of solid oxide fuel cells by sacrificial template methods. Renew. Sustain. Energy Rev..

[34] Liu X, Liu X, Sun B, Zhou H, Fu A, Wang Y, Guo Y-G, Guo P, Li H (2018). Carbon materials with hierarchical porosity: effect of template removal strategy and study on their electrochemical properties. Carbon.

[35] Scotti KL, Dunand DC (2018). Freeze casting—a review of processing, microstructure and properties via the open data repository FreezeCastingnet. Progr. Mater. Sci..

[36] Hwa Y, Yi E, Shen H, Sung Y, Kou J, Chen K, Parkinson DY, Doeff MM, Cairns EJ (2019). Three-dimensionally aligned sulfur electrodes by directional freeze tape casting. Nano Lett..

[37] Yi E, Shen H, Heywood S, Alvarado J, Parkinson DY, Chen G, Sofie SW, Doeff MM (2020). All-solid-state batteries using rationally designed garnet electrolyte frameworks. ACS Appl. Energy Mater..

[38] Chen C, Zhang Y, Li Y, Kuang Y, Song J, Luo W, Wang Y, Yao Y, Pastel G, Xie J, Hu L (2017). Highly conductive, lightweight, low-tortuosity carbon frameworks as ultrathick 3D current collectors. Adv. Energy Mater..

[39] Kong L, Peng H-J, Huang J-Q, Zhang Q (2017). Review of nanostructured current collectors in lithium–sulfur batteries. Nano Res..

[40] Jin S, Jiang Y, Ji H, Yu Y (2018). Advanced 3D current collectors for lithium-based batteries. Adv. Mater..

[41] Yue Y, Liang H (2018). 3D current collectors for lithium-ion batteries: a topical review. Small Methods.

[42] Ion JC, Ion JC (2005). Chapter 5—Engineering materials. laser processing of engineering materials.

[43] Shirazi SFS, Gharehkhani S, Mehrali M, Yarmand H, Metselaar HSC, Adib Kadri N, Osman NAA (2015). A review on powder-based additive manufacturing for tissue engineering: selective laser sintering and inkjet 3D printing. Sci. Technol. Adv. Mater..

[44] Daly R, Harrington TS, Martin GD, Hutchings IM (2015). Inkjet printing for pharmaceutics—a review of research and manufacturing. Int. J. Pharm..

[45] Derby B (2015). Additive manufacture of ceramics components by inkjet printing. Engineering.

[46] Melchels FPW, Feijen J, Grijpma DW (2010). A review on stereolithography and its applications in biomedical engineering. Biomaterials.

[47] Yang Y, Li L (2018). Cost modeling and analysis for mask image projection stereolithography additive manufacturing: simultaneous production with mixed geometries. Int. J. Prod. Econ..

[48] Halloran JW (2016). Ceramic stereolithography: additive manufacturing for ceramics by photopolymerization. Annu. Rev. Mater. Res..

[49] Serdeczny MP, Comminal R, Pedersen DB, Spangenberg J (2018). Experimental validation of a numerical model for the strand shape in material extrusion additive manufacturing. Addit. Manuf..

[50] Gonzalez-Gutierrez J, Cano S, Schuschnigg S, Kukla C, Sapkota J, Holzer C (2018). Additive manufacturing of metallic and ceramic components by the material extrusion of highly-filled polymers: a review and future. Perspectives.

[51] Khairallah SA, Anderson AT, Rubenchik A, King WE (2016). Laser powder-bed fusion additive manufacturing: physics of complex melt flow and formation mechanisms of pores, spatter, and denudation zones. Acta Mater..

[52] King WE, Anderson AT, Ferencz RM, Hodge NE, Kamath C, Khairallah SA, Rubenchik AM (2015). Laser powder bed fusion additive manufacturing of metals; physics, computational, and materials challenges. Appl. Phys. Rev..

[53] Guan X, Zhao YF (2020). Modeling of the laser powder-based directed energy deposition process for additive manufacturing: a review. Int. J. Adv. Manuf. Technol..

[54] Saboori A, Aversa A, Marchese G, Biamino S, Lombardi M, Fino P (2019). Application of directed energy deposition-based additive manufacturing in repair. Appl. Sci..

[55] Xiong G, Meng C, Reifenberger RG, Irazoqui PP, Fisher TS (2014). A review of graphene-based electrochemical microsupercapacitors. Electroanalysis.

[56] Park JB, Xiong W, Gao Y, Qian M, Xie ZQ, Mitchell M, Zhou YS, Han GH, Jiang L, Lu YF (2011). Fast growth of graphene patterns by laser direct writing. Appl. Phys. Lett..

[57] Delaporte P, Alloncle A-P (2016). Laser-induced forward transfer: a high resolution additive manufacturing technology. Opt. Laser Technol..

[58] Serra P, Piqué A (2019). Laser-induced forward transfer: fundamentals and applications. Adv. Mater. Technol..

[59] Chatham CA, Long TE, Williams CB (2019). A review of the process physics and material screening methods for polymer powder bed fusion additive manufacturing. Prog. Polym. Sci..

[60] Ibrahim KA, Wu B, Brandon NP (2016). Electrical conductivity and porosity in stainless steel 316L scaffolds for electrochemical devices fabricated using selective laser sintering. Mater. Des..

[61] Lahtinen E, Kukkonen E, Jokivartio J, Parkkonen J, Virkajärvi J, Kivijärvi L, Ahlskog M, Haukka M (2019). Preparation of highly porous carbonous electrodes by selective laser sintering. ACS Appl. Energy Mater..

[62] Chen W-S, Ho H-J (2018). Recovery of valuable metals from lithium-ion batteries NMC cathode waste materials by hydrometallurgical methods. Metals.

[63] Deiner LJ, Jenkins T, Powell A, Howell T, Rottmayer M (2019). High capacity rate capable aerosol jet printed Li-ion battery cathode. Adv. Eng. Mater..

[64] Katherine AA, Dupuy AD, Bertoli US, Zheng B, West WC, Chen QN, Shapiro AA, Schoenung JM (2021). Morphology, microstructure, and phase states in selective laser sintered lithium ion battery cathodes. J. Mater. Process. Technol..

[65] Iwabuchi Y, Yan J (2015). Laser sintering of silicon powder and carbon nanofibers for porous composite thick films. Appl. Phys. Express.

[66] Yu Y, Chen M, Wang S, Hill C, Joshi P, Kuruganti T, Hu A (2018). Laser sintering of printed anodes for Al-air batteries. J. Electrochem. Soc..

[67] Chee WK, Lim HN, Zainal Z, Huang NM, Harrison I, Andou Y (2016). Flexible graphene-based supercapacitors: a review. J. Phys. Chem. C.

[68] Tan RKL, Reeves SP, Hashemi N, Thomas DG, Kavak E, Montazami R, Hashemi NN (2017). Graphene as a flexible electrode: review of fabrication approaches. J. Mater. Chem. A.

[69] Jiang W, Wang H, Xu Z, Li N, Chen C, Li C, Li J, Lv H, Kuang L, Tian X (2018). A review on manifold synthetic and reprocessing methods of 3D porous graphene-based architecture for Li-ion anode. Chem. Eng. J..

[70] Al Hassan MR, Sen A, Zaman T, Mostari MS (2019). Emergence of graphene as a promising anode material for rechargeable batteries: a review. Mater. Today Chem..

[71] Ye R, James DK, Tour JM (2018). Laser-induced graphene. Acc. Chem. Res..

[72] Wan Z, Streed EW, Lobino M, Wang S, Sang RT, Cole IS, Thiel DV, Li Q (2018). Laser-reduced graphene: synthesis, properties, and applications. Adv. Mater. Technol..

[73] Ye R, James DK, Tour JM (2019). Laser-induced graphene: from discovery to translation. Adv. Mater..

[74] Kurra N, Jiang Q, Nayak P, Alshareef HN (2019). Laser-derived graphene: a three-dimensional printed graphene electrode and its emerging applications. Nano Today.

[75] Lavagna L, Meligrana G, Gerbaldi C, Tagliaferro A, Bartoli M (2020). Graphene and lithium-based battery electrodes: a review of recent literature. Energies.

[76] Li G (2020). Direct laser writing of graphene electrodes. J. Appl. Phys..

[77] Zhang F, Alhajji E, Lei Y, Kurra N, Alshareef HN (2018). Highly doped 3D graphene Na-ion battery anode by laser scribing polyimide films in nitrogen ambient. Adv. Energy Mater..

[78] Ren M, Zhang J, Fan M, Ajayan PM, Tour JM (2019). Li-breathing air batteries catalyzed by MnNiFe/laser-induced graphene catalysts. Adv. Mater..

[79] Alhajji E, Wang W, Zhang W, Alshareef HN (2020). A hierarchical three-dimensional porous laser-scribed graphene film for suppressing polysulfide shuttling in lithium–sulfur batteries. ACS Appl. Mater. Interfaces.

[80] Ren M, Zhang J, Tour JM (2018). Laser-induced graphene synthesis of Co_3_O_4_ in graphene for oxygen electrocatalysis and metal-air batteries. Carbon.

[81] Osgood H, Devaguptapu SV, Xu H, Cho J, Wu G (2016). Transition metal (Fe Co, Ni, and Mn) oxides for oxygen reduction and evolution bifunctional catalysts in alkaline media. Nano Today.

[82] Cheng X-B, Zhang R, Zhao C-Z, Zhang Q (2017). Toward safe lithium metal anode in rechargeable batteries: a review. Chem. Rev..

[83] Xu X, Wang S, Wang H, Xu B, Hu C, Jin Y, Liu J, Yan H (2017). The suppression of lithium dendrite growth in lithium sulfur batteries: a review. J. Energy Storage.

[84] Zhao H, Deng N, Yan J, Kang W, Ju J, Ruan Y, Wang X, Zhuang X, Li Q, Cheng B (2018). A review on anode for lithium-sulfur batteries: progress and prospects. Chem. Eng. J..

[85] Yi J, Chen J, Yang Z, Dai Y, Li W, Cui J, Ciucci F, Lu Z, Yang C (2019). Facile patterning of laser-induced graphene with tailored Li nucleation kinetics for stable lithium-metal batteries. Adv. Energy Mater..

[86] Chen W, Salvatierra RV, Ren M, Chen J, Stanford MG, Tour JM (2020). Laser-induced silicon oxide for anode-free lithium metal batteries. Adv. Mater..

[87] Song M-K, Cairns EJ, Zhang Y (2013). Lithium/sulfur batteries with high specific energy: old challenges and new opportunities. Nanoscale.

[88] Pang Q, Liang X, Kwok CY, Nazar LF (2016). Advances in lithium–sulfur batteries based on multifunctional cathodes and electrolytes. Nat. Energy.

[89] Wei M, Zhang F, Wang W, Alexandridis P, Zhou C, Wu G (2017). 3D direct writing fabrication of electrodes for electrochemical storage devices. J. Power Sources.

[90] Nayak L, Mohanty S, Nayak SK, Ramadoss A (2019). A review on inkjet printing of nanoparticle inks for flexible electronics. J. Mater. Chem. C.

[91] Sopeña P, Arrese J, González-Torres S, Fernández-Pradas JM, Cirera A, Serra P (2017). Low-cost fabrication of printed electronics devices through continuous wave laser-induced forward transfer. ACS Appl. Mater. Interfaces.

[92] Wartena R, Curtright AE, Arnold CB, Piqué A, Swider-Lyons KE (2004). Li-ion microbatteries generated by a laser direct-write method. J. Power Sources.

[93] A. Pique, C. Arnold, R. Wartena, D. Weir, B. Pratap, K. Swider-Lyons, R. Kant, D. Chrisey, Laser-induced forward transfer direct-write of miniature sensor and microbattery systems, in *Proceeding SPIE*, 4830 (2003).

[94] A. Atre, C. Arnold, LiCoO_2_ texturing by laser induced forward transfer for printed microbatteries, in *Proceeding SPIE*, 7921 (2011).

[95] Kim H, Auyeung RCY, Piqué A (2007). Laser-printed thick-film electrodes for solid-state rechargeable Li-ion microbatteries. J. Power Sources.

[96] Arnold CB, Kim H, Piqué A (2004). Laser direct write of planar alkaline microbatteries. Appl. Phys. A.

[97] Bates JB, Dudney NJ, Lubben DC, Gruzalski GR, Kwak BS, Yu X, Zuhr RA (1995). Thin-film rechargeable lithium batteries. J. Power Sources.

[98] Humble PH, Harb JN, LaFollette R (2001). Microscopic nickel-zinc batteries for use in autonomous microsystems. J. Electrochem. Soc..

[99] Rosenberg S, Hintennach A (2015). In situ formation of α-MnO_2_ nanowires as catalyst for sodium-air batteries. J. Power Sources.

[100] Constantinescu C, Vizireanu S, Ion V, Aldica G, Stoica SD, Lazea-Stoyanova A, Alloncle A-P, Delaporte P, Dinescu G (2016). Laser-induced forward transfer of carbon nanowalls for soft electrodes fabrication. Appl. Surf. Sci..

[101] Li G, Mo X, Law W-C, Chan KC (2019). 3D printed graphene/nickel electrodes for high areal capacitance electrochemical storage. J. Mater. Chem. A.

[102] Miller JC (1994). Laser ablation: principles and applications.

[103] Kuwata N, Kumar R, Toribami K, Suzuki T, Hattori T, Kawamura J (2006). Thin film lithium ion batteries prepared only by pulsed laser deposition. Solid State Ionics.

[104] Makimura Y, Rougier A, Tarascon J-M (2006). Pulsed laser deposited iron fluoride thin films for lithium-ion batteries. Appl. Surf. Sci..

[105] Park MS, Wang GX, Liu HK, Dou SX (2006). Electrochemical properties of Si thin film prepared by pulsed laser deposition for lithium ion micro-batteries. Electrochim. Acta.

[106] Cui Y-H, Xue M-Z, Wang X-L, Hu K, Fu Z-W (2009). InP as new anode material for lithium ion batteries. Electrochem. Commun..

[107] Sakuda A, Hayashi A, Ohtomo T, Hama S, Tatsumisago M (2011). All-solid-state lithium secondary batteries using LiCoO2 particles with pulsed laser deposition coatings of Li_2_S–P_2_S_5_ solid electrolytes. J. Power Sources.

[108] Sakurai Y, Sakuda A, Hayashi A, Tatsumisago M (2011). Preparation of amorphous Li_4_SiO_4_–Li_3_PO_4_ thin films by pulsed laser deposition for all-solid-state lithium secondary batteries. Solid State Ionics.

[109] Lin Z, Ye X, Han J, Chen Q, Fan P, Zhang H, Xie D, Zhu H, Zhong M (2015). Precise control of the number of layers of graphene by picosecond laser thinning. Sci. Rep..

[110] Lutey AHA, Fortunato A, Ascari A, Carmignato S, Leone C (2015). Laser cutting of lithium iron phosphate battery electrodes: characterization of process efficiency and quality. Opt. Laser Technol..

[111] Lee D, Ahn S (2017). Investigation of laser cutting width of LiCoO_2_ coated aluminum for lithium-ion batteries. Appl. Sci..

[112] Lee D (2018). Investigation of physical phenomena and cutting efficiency for laser cutting on anode for Li-ion batteries. Appl. Sci..

[113] Zhang N, Zheng Y, Trifonova A, Pfleging W (2017). Laser structured Cu foil for high-performance lithium-ion battery anodes. J. Appl. Electrochem..

[114] Habedank J, Endres J, Schmitz P, Zaeh M, Huber H (2018). Femtosecond laser structuring of graphite anodes for improved lithium-ion batteries: ablation characteristics and process design. J. Laser Appl..

[115] Habedank JB, Günter FJ, Billot N, Gilles R, Neuwirth T, Reinhart G, Zaeh MF (2019). Rapid electrolyte wetting of lithium-ion batteries containing laser structured electrodes: in situ visualization by neutron radiography. Int. J. Adv. Manuf. Technol..

[116] Kim Y, Drews A, Chandrasekaran R, Miller T, Sakamoto J (2018). Improving Li-ion battery charge rate acceptance through highly ordered hierarchical electrode design. Ionics.

[117] Park J, Hyeon S, Jeong S, Kim H-J (2019). Performance enhancement of Li-ion battery by laser structuring of thick electrode with low porosity. J. Ind. Eng. Chem..

[118] Su Y, Tong R-A, Zhang H, Liang P, Wang C-A, Zhong M (2019). Defocused laser ablation process—a high-efficiency way to fabricate MoO_3_—Mo integrative anode with excellent electrochemical performance for lithium ion batteries. J. Alloys Compd..

[119] R. Kohler, J. Proell, S. Ulrich, V. Trouillet, S. Indris, M. Przybylski, W. Pfleging, Laser-assisted structuring and modification of LiCoO_2_ thin films, in *Proceeding SPIE*, 7202 (2009).

[120] Kohler R, Smyrek P, Ulrich S, Bruns M, Trouillet V, Pfleging W (2010). Materials, patterning and annealing of nanocrystalline LiCoO_2_ thin films. J. Optoelectr. Adv. Mater..

[121] J. Pröll, R. Kohler, C. Adelhelm, M. Bruns, M. Torge, S. Heißler, M. Przybylski, C. Ziebert, W. Pfleging, Laser modification and characterization of Li-Mn-O thin film cathodes for lithium-ion batteries, in *Proceeding SPIE*, 7921 (2011).

[122] Pröll J, Kohler R, Torge M, Ulrich S, Ziebert C, Bruns M, Seifert HJ, Pfleging W (2011). Laser microstructuring and annealing processes for lithium manganese oxide cathodes. Appl. Surf. Sci..

[123] Proell J, Kohler R, Mangang A, Ulrich S, Ziebert C, Pfleging W (2012). 3D structures in battery materials. J. Laser Micro Nanoeng..

[124] Pröll J, Kohler R, Mangang A, Ulrich S, Bruns M, Seifert HJ, Pfleging W (2012). Diode laser heat treatment of lithium manganese oxide films. Appl. Surf. Sci..

[125] Kohler R, Proell J, Bruns M, Ulrich S, Seifert HJ, Pfleging W (2013). Conical surface structures on model thin-film electrodes and tape-cast electrode materials for lithium-ion batteries. Appl. Phys. A.

[126] Pröll J, Weidler PG, Kohler R, Mangang A, Heißler S, Seifert HJ, Pfleging W (2013). Comparative studies of laser annealing technique and furnace annealing by X-ray diffraction and Raman analysis of lithium manganese oxide thin films for lithium-ion batteries. Thin Solid Films.

[127] W. Pfleging, R. Kohler, J. Pröll, Laser generated microstructures in tape cast electrodes for rapid electrolyte wetting: new technical approach for cost efficient battery manufacturing, in *Proceeding SPIE*, 8968 (2014).

[128] Kim JS, Pfleging W, Kohler R, Seifert HJ, Kim TY, Byun D, Jung H-G, Choi W, Lee JK (2015). Three-dimensional silicon/carbon core–shell electrode as an anode material for lithium-ion batteries. J. Power Sources.

[129] Park C-M, Kim J-H, Kim H, Sohn H-J (2010). Li-alloy based anode materials for Li secondary batteries. Chem. Soc. Rev..

[130] Smyrek P, Pröll J, Seifert HJ, Pfleging W (2015). Laser-induced breakdown spectroscopy of laser-structured Li(NiMnCo)O_2_ electrodes for lithium-ion batteries. J. Electrochem. Soc..

[131] R. Kohler, M. Bruns, P. Smyrek, S. Ulrich, M. Przybylski, W. Pfleging, Laser annealing of textured thin film cathode material for lithium ion batteries, in *Proceeding SPIE*, 8968 (2010).

[132] Kohler R, Smyrek P, Ulrich S, Bruns M, Trouillet V, Pfleging W (2010). Patterning and annealing of nanocrystalline LiCoO_2_ thin films. J. Optoelectron. Adv. Mater..

[133] Pfleging W, Pröll J (2014). A new approach for rapid electrolyte wetting in tape cast electrodes for lithium-ion batteries. J. Mater. Chem. A.

